# Everything fine so far? Physical and mental health in HIV-infected patients with virological success and long-term exposure to antiretroviral therapy

**DOI:** 10.7448/IAS.17.4.19673

**Published:** 2014-11-02

**Authors:** Gesa Erdbeer, Michael Sabranski, Ina Sonntag, Albrecht Stoehr, Heinz-A. Horst, Andreas Plettenberg, Knud Schewe, Stefan Unger, Hans-J. Stellbrink, Stefan Fenske, Christian Hoffmann

**Affiliations:** 1 University of Schleswig-Holstein, Campus Kiel, Kiel, Germany; 2 Infektionsmedizinisches Centrum Hamburg, Study Center, Hamburg, Germany; 3 IFI, Institute for Interdisciplinary Medicine, Hamburg, Germany

## Abstract

**Introduction:**

Little is known about the well-being on long-term exposure to antiretroviral therapy. The ACTG Augmented Symptoms Distress Module (ASDM) is a validated tool which measures the presence of a total of 22 symptoms seen with HIV and quantifies the extent to which they cause distress to the patient.

**Methods:**

ELBE was a cross-sectional study that consecutively included adult HIV-infected patients presenting with viral suppression (<50 HIV RNA copies/mL) and ART exposure for at least five years. Patients were evaluated by four different questionnaires, including ASDM.

**Results:**

Of a total of 894 patients included in the three participating ELBE centres, complete data on ASDM were available for 698 patients (626 male, 69 female, 3 transsexual). Median age was 49.7 years (range, 23.3–82.5 years) and median exposure to ART was 11.5 years (range, 5–28 years). Median CD4 T-cell counts had increased from a CD4 nadir of 180 to currently 640 cells/µL. Despite immunological and virological success, a high degree of symptom-related distress was noted in this patient population. In total, 63.8% and 36.3% of the patients had at least one “bothersome” or one “very bothersome” symptom, respectively. The symptoms most frequently reported to be “bothersome” or “very bothersome” were fatigue and energy loss (18.5% and 11.0% respectively), insomnia (12.8% and 11.6%), sadness and depression (13.0% and 10.0%), sexual dysfunction (12.0% and 10.0%), and changes in body appearance (11.0% and 10.9%). There was no association between the degree of symptom-related distress and gender, age or CD4 T-cell nadir. However, the history of AIDS-defining illnesses, comorbidities such as depression but also the duration of ART were significantly associated with a higher overall symptom summary score and with a higher frequency of symptoms. For example, in patients with at least 15 years of ART exposure, only 27.3% of the patients did not report at least one “bothersome” or “very bothersome” symptom.

**Conclusions:**

In this large group of positively selected HIV+ patients with virological success and long-term exposure to ART, a high degree of symptom-related distress was found. Medical care of HIV-infected patients should not only focus on optimal virological outcome. More data on quality of life in patients with long-term exposure to ART is needed.

